# Mechanisms of fibrosis and novel antifibrotic therapies

**DOI:** 10.1016/j.ero.2026.100237

**Published:** 2026-09-10

**Authors:** Jörg H.W. Distler

**Affiliations:** 1 Department of Rheumatology, University Hospital Düsseldorf, Heinrich-Heine University, Düsseldorf, Germany; 2 Hiller Research Center, University Hospital Düsseldorf, Heinrich-Heine University, Düsseldorf, Germany

## PATHOPHYSIOLOGY OF FIBROTIC TISSUE REMODELLING

Fibrosis in systemic sclerosis (SSc) is initiated by an unknown trigger that leads to endothelial cell injury and activation of the immune system with type 2-biased immune responses and prominent B cell activation and autoimmunity [1]. Microvascular injury and autoimmunity promote activation of fibroblasts as key effector cells of fibrotic remodelling. These activated fibroblasts are a heterogenous population of cells with functionally distinct subpopulations and can originate from multiple cell types, including tissue-resident fibroblasts, pericytes, endothelial cells, and potentially also epithelial cells, tissue-resident precursor cells, and fibrocytes [2]. These fibroblasts produce excessive extracellular matrix, leading to progressive fibrotic remodelling of the tissues [3]. In addition, activated fibroblast subpopulations release proinflammatory and vasoactive mediators, thus sustaining a vicious cycle of inflammation, vascular damage, and fibrosis.

## SPATIAL OMICS-BASED IDENTIFICATION OF NOVEL FIBROBLAST SUBPOPULATIONS

Recent advancements in spatial OMICs, including imaging mass cytometry (IMC) as a multiplexed proteomic approach and cyclic *in situ* hybridisation (cISH) as a transcriptomic approach, have enabled identification of novel disease-associated fibroblast subpopulations [[4], [5], [6]].

IMC identified multiple distinct subpopulations of fibroblasts in the skin: ADAM12^+^GLI1^+^, myofibroblasts, resting fibroblasts, ADRP^+^, CDH11^+^, FAP^high^, PI16^+^FAP^−^, PI16^+^FAP^+^, PI16^+^FAP^+^TFAM^low^, S1PR^+^, TFAM^high^, THY1+ADAM12^high^PU.1^high^, and THY1^+^ADAM12^low^ fibroblast subpopulations [6]. Several of these populations are changed in frequency in SSc skin compared to normal skin, including upregulation of ADAM12^+^GLI1^+^, myofibroblasts, FAP^high^, S1PR^+^, and PU.1^high^THY1+ADAM12^high^ fibroblasts and downregulation of PI16^+^FAP^+^, TFAM^high^, and THY1^+^ADAM12^low^ fibroblasts. In addition to changes in frequencies, the spatial organisation of fibroblasts and the cellular interactions of fibroblasts are altered in SSc [6]. cISH-based analyses confirmed shifts in the frequencies of fibroblast subpopulations, changes in their topographical interactions, and enabled further characterisation of transcriptional profiles of individual fibroblast subpopulations [4].

## CLINICAL DIAGNOSTICS AND MOLECULAR IMAGING

The identification of specific fibroblast subpopulations also offers new avenues for clinical diagnostics. Molecular imaging of fibroblast activation protein (FAP) using FAP inhibitors labelled with radioactive tracers (FAPI) may serve as an imaging biomarker for quantifying fibroblast activation and thereby provide an opportunity to quantify fibrotic activity. Patients with SSc-related interstitial lung disease (ILD) demonstrate significantly higher FAPI uptake in the lungs compared to controls. The areas of FAPI accumulation map to areas of fibrosis on high-resolution computed tomography [7]. Of note, patients with progression of ILD on follow-up showed higher FAPI uptake at baseline than those with stable disease, suggesting that FAPI-based imaging can function as a marker for fibrotic activity. FAPI has also been shown to accumulate in other organs undergoing fibrotic remodelling in SSc such as the heart [8].

## THERAPEUTIC TARGETING OF FIBROBLAST SUBPOPULATIONS

The identification of novel, functionally distinct subpopulations of fibroblasts may also yield novel targets for therapeutic intervention. This is exemplified by PU.1^high^THY1+ADAM12^high^ fibroblasts and targeting of the upstream transcription factor PU.1 [3]. PU.1 is a member of the E26 transformation-specific (ETS) family of transcription factors that is upregulated in SSc [9]. Forced overexpression of PU-1 is sufficient to induce differentiation of resting fibroblasts into activated, myofibroblast-like cells and can convert inflammatory fibroblasts, eg, isolated from inflamed synovial tissue of patients with rheumatoid arthritis, into profibrotic myofibroblasts [9]. Knockdown of PU.1 inhibits fibroblast activation in cultured fibroblasts and in organotypic models. Moreover, inducible knockout of PU.1 in fibroblasts or treatment with PU.1 inhibitors prevents fibrotic tissue remodelling in murine models of dermal, pulmonary, and hepatic fibrosis [9].

## OMICS-BASED ASSESSMENT OF NOVEL THERAPEUTICS

OMICs approaches are also utilised to assess the effects of novel therapeutics, such as CD19-targeted Chimeric antigen receptor T cells (CAR-T) cell therapy, with cellular and spatial mapping of molecular responses. In SSc patients treated with CD19.CAR-T cells, repeated skin biopsies taken over a 12-month follow-up period show progressive normalisation of skin histology, providing evidence for tissue regeneration [10]. Analysis of the fibroblast composition reveals a shift towards a healthy-like profile, including a decrease in PU.1^high^THY1+ADAM12^high^ fibroblasts and myofibroblasts [10].

## BISPECIFIC T CELL ENGAGERS IN REFRACTORY DISEASE

Bispecific T cell engagers (TCEs) represent a therapeutic alternative for deep B cell depletion in refractory fibrosing connective tissue diseases.

### BCMA-TCE (teclistamab) in SSc

In a study of 5 patients with severe, treatment-refractory SSc who had failed multiple prior therapies, induction therapy with the B-cell maturation antigen (BCMA)-TCE teclistamab followed by maintenance therapy with rituximab resulted in significant clinical improvements [11]. All patients experienced a major reduction in the modified Rodnan Skin Score, alongside declines in tendon friction rubs and the European ccleroderma trials and research group (EUSTAR) Activity Score and stabilisation of SSc-ILD. Immunologically, the treatment led to near-complete depletion of BCMA^+^ cells in skin biopsies. Serologically, anti-Scl70 autoantibody levels were markedly reduced and became undetectable with longer follow-up, along with major decreases in total immunoglobulin (Ig) G, IgA, and IgM levels [11]. However, treatment with teclistamab was associated with cytokine release syndrome (up to grade 3) and infections.

### CD19-TCE (blinatumomab) in ASyS

Similarly, the CD19-TCE blinatumomab was evaluated in 5 patients with treatment-refractory antisynthetase syndrome (ASyS) [11]. Induction therapy with blinatumomab followed by maintenance therapy with rituximab resulted in the normalisation of creatine kinase levels and substantial improvements in the 6-minute walking distance for all patients. ILD stabilised or improved. Muscle biopsies confirmed marked reduction in CD19 counts, showed a marked reduction in inflammation (CD8^+^, CD163^+^, MHC-I, CD3^+^, and C5b-9), and provided evidence of muscle regeneration [11].

## CONCLUSION

Novel OMICs approaches are essential for identifying and characterising new cellular subsets, providing deeper insights into the pathophysiology of fibrotic diseases. These technologies enable the assessment of molecular responses to novel therapeutics, offer opportunities to refine diagnostics, and identify potential targets for more specific antifibrotic therapies. Furthermore, deep B cell depletion using TCEs emerges as a highly promising novel option for the treatment of fibrosing autoimmune diseases.

## CRediT authorship contribution statement

**Jörg H.W. Distler:** Conceptualization, Investigation, Writing – original draft, Writing – review & editing.
